# Spectroscopic Features of Weak Intermolecular Interactions: IR Spectrum of the Formamide–Water Complex Revisited

**DOI:** 10.1021/acs.jpca.6c02029

**Published:** 2026-07-31

**Authors:** Justyna Krupa, Silvia Alessandrini, Cristina Puzzarini, Malgorzata Biczysko

**Affiliations:** † Faculty of Chemistry, 49572 University of Wroclaw, F. Joliot-Curie 14, 50-383 Wroclaw, Poland; ‡ Dipartimento di Chimica “Giacomo Ciamician”, 9296 Università di Bologna, Via P. Gobetti 85, 40129 Bologna, Italy

## Abstract

This work provides reference data on the vibrational spectrum of formamide and its modification upon complexation with one water molecule, relevant for the interpretation of complex spectral patterns observed in interstellar ices both in the laboratory experiment and with the James Webb Space Telescope. To this end, new measurements of the formamide and formamide–water vibrational spectra have been performed using low-temperature argon matrix isolation fourier transform infrared (MI-FTIR) spectroscopy. Analysis and interpretation of experimental spectra have been supported by fully anharmonic vibrational second-order perturbation theory computations, which allowed the assignment of additional nonfundamental bands of formamide as well as for unveiling an elusive formamide–water structure. This intermolecular complex, characterized by an O···H–O hydrogen bond, had not been identified in prior MI-FTIR studies, although it was observed in the gas phase.

## Graphical abstract

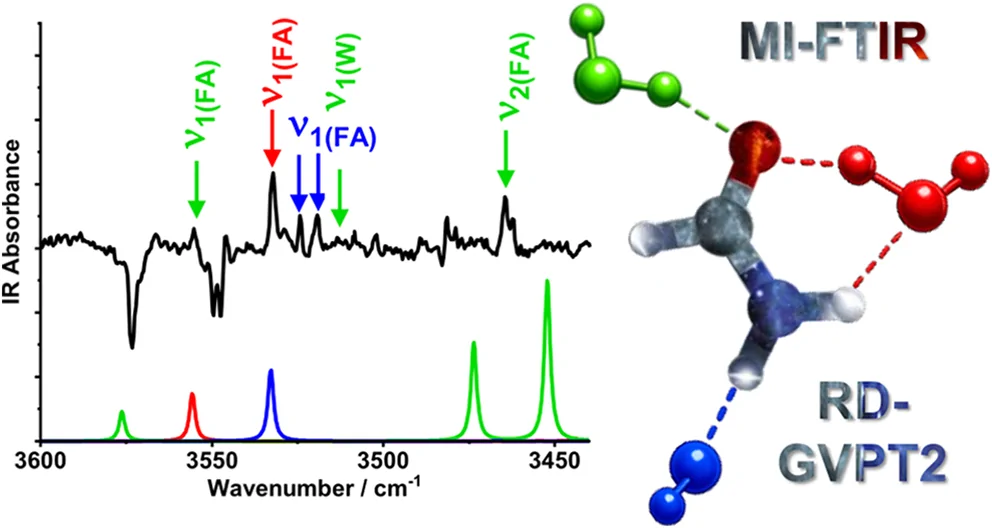

## Introduction

Interstellar ices, which are mainly composed of small inorganic molecules such as H_2_O, CO, CO_2_, NH_3_, and CH_3_OH, coat dust grains and constitute microscopic reaction environments where simple C-, H-, N-, and O-bearing species reside, aggregate, and react.^1–10^ Ultimately, they are considered the primary environment where the so-called “complex organic molecules” (COMs),^11^ often having a prebiotic character, are formed.^11^ The composition and chemical reactivity of such ice mixtures are currently being probed by the James Webb Space Telescope (JWST),^3,7,12,13^ primarily in the infrared (IR) spectral range. Infrared bands of the ices are particularly sensitive to intermolecular interactions, molecular aggregation, and the local ice environment. Therefore, interpreting these features and fully understanding them remain challenging tasks that require a strong interplay between theoretical and experimental studies.^14^

Among molecules potentially present in the ices, formamide (NH_2_CHO, FA) represents the simplest amide and, due to its NCO backbone, is considered as a key species in prebiotic chemistry.^1,15^ Formamide has been observed in several astronomical environments, from the interstellar medium,^16–18^ to comets,^19^ and satellites.^20^ Its presence is so ubiquitous that a theory of the origin of life based on such species has been suggested.^15^ Indeed, formamide can be connected to a large series of biologically relevant molecules, and it is considered a relevant molecule in the Miller-Urey experiment.^15,21,22^ Laboratory studies also suggest that UV irradiation of formamide-containing ices leads to the formation of glycine and related species,^23^ and that the ice composition plays a primary role in both the destruction of the deposited species and the formation of new compounds.^24^ It is interesting to note that, although detected in the gas phase, formamide has never been found in ices by JWST observations.^25^ Indeed, several formamide diagnostic bands^26^ overlap strongly with features of water- and methanol-rich ices, so that JWST IR spectra can only provide upper limits rather than firm detections.^7,13,25,27^ The difficulty of assigning IR spectra in ices is exemplified by the fact that the most intense and characteristic IR feature of formaldehyde, arising from the C=O stretching mode and commonly used for its identification among more abundant species, is strongly influenced by interactions within the ice environment.^14,25,28^

Weak intermolecular interactions, such as hydrogen bonding with surrounding water molecules, can also affect photon-initiated processes. In fact, the environment can affect how absorbed energy is redistributed via intramolecular vibrations in the ground or excited electronic states,^29^ thus modulating photolysis pathways and their efficiencies.^30–33^ It is thus crucial to understand through the interplay of experiment and theory how the ice composition affects the photoprocessing of simple prebiotic molecules, such as formamide. This would provide a crucial step toward understanding their IR signatures and linking them to the photochemical behavior in astrophysical ices.

In this respect, Matrix-Isolation Fourier Transform Infrared (MI-FTIR) experiments on molecular aggregates complemented by state-of-the-art quantum chemical calculations^34–38^ allow one to gain insights into the specific interaction-driven spectral features that are necessary to dissect the complex spectral patterns of multicomponent ices. Within the matrix-isolation approach, argon matrix at cryogenic temperatures effectively suppresses translational and rotational motion, thus yielding high-resolution, narrow-bandwidth IR vibrational spectra dominated by fundamental transitions.^39–41^ Under sufficiently high dilution, guest molecules are statistically separated in the inert host matrix, minimizing intermolecular interactions and enabling spectra closely resembling those of isolated monomers. In contrast, increasing the guest-to-host ratio (i.e., the dopant concentration in the gas mixture prior to codeposition) promotes controlled intermolecular encounters and the formation of weakly bound aggregates, including dimers, trimers, higher clusters, and specific molecular complexes. In the MI-FTIR spectra, the molecular structures and compositions of complexes are deduced, based on indirect evidence, from the vibrational patterns, namely perturbation of the vibrational states of monomers and/or appearance of new bands.^34–38,42^

In this context, codeposition of formamide and water leads to the formation of formamide–water adducts and offers a laboratory proxy for local environments in interstellar ices, where hydrogen bonding and the surrounding ice environment are expected to reshape diagnostic vibrational signatures, particularly in the N–H, O–H, and C=O stretching regions. At the same time, weak matrix-induced perturbations, such as small frequency shifts and site splitting arising from different trapping sites, must be carefully distinguished from real complexation effects. The intermolecular interactions between formamide and water are dominated by hydrogen bonds (HBs). Three 1:1 and one 1:2 formamide–water (FAW) adducts have been characterized in jet-expansion rotational spectroscopy experiments,^43,44^ with larger clusters also later observed in the gas phase.^45,46^ Differently, only two 1:1 FAW complexes have been identified in matrix isolation environment.^34,47^ Although formamide has been extensively investigated in noble-gas matrices,^35,48–50^ FAW complexes have been characterized only in a limited number of matrix-isolation studies.^34,47^ In this work, we aim at filling this gap by combining new MI-FTIR measurements with state-of-the-art vibrational second-order perturbation theory (VPT2)^51–54^ computations, thus providing an improved interpretation of the IR fingerprints of the FAW adducts dominated by weak intermolecular interactions. The computational investigation, based on density functional theory (DFT), of structural and spectroscopic properties, has also allowed us to highlight specific interaction patterns. For this purpose, selected hybrid and double-hybrid DFT functionals in conjunction with an efficient basis set have been considered to assess the accuracy of methodologies applicable to larger molecular aggregates mimicking interstellar ice micrograins.

In the following, the experimental methodology will be described together with the computational strategy employed to support the assignments of the experimental features. Then, the results section critically presents the outcomes of this study. First, we focus on formamide and, then, we turn our attention to the structural and spectroscopic properties of the FAW complexes. Finally, the discussion on experimental and computational strategies applicable to larger and more complex molecular interstellar ice analogues will be reported.

## Methodology

### Experimental Details

Formamide (FA, 99%) and argon (purity 5.0) were purchased from Sigma-Aldrich (Schnelldorf Distribution) and Linde (Dublin, Ireland), respectively. Prior to the experiment, FA was dried over molecular sieves and subjected to repeated freeze–pump–thaw cycles to remove volatile impurities. The purified liquid FA was evaporated from a small glass bulb maintained at 250 K and positioned outside the cryostat chamber. The FA vapor was mixed with a large excess of Ar (for the monomer) or with a H_2_O/Ar gas mixture (H_2_O/Ar = 1:1000) inside the cryostat chamber, at a distance of approximately 5 cm from the coldfinger, and immediately codeposited onto a CsI window held at 15 K. The deposited samples were subsequently annealed at 30 K for 5 min. The H_2_O/Ar mixtures were prepared in a stainless-steel vacuum line by premixing water vapor with argon. Although the absolute concentration of FA in the matrices can not be exactly determined, it was controlled qualitatively by adjusting the pressure of the deposited matrix gas. The pressure and deposition rate of the gas mixture were monitored and regulated using piezoelectric transducers (MKS Instruments, model 902B, Andover, MA, USA; accuracy ±1%) installed in the deposition line. A typical deposition rate of 2 mbar·min^–1^ was used. The total amount of deposited gas was approximately 150 mbar. Deposition conditions were optimized to yield matrices containing predominantly monomeric FA and its 1:1 complexes. The low temperature was obtained using a closed cycle helium cryostat (APDCryogenics, Macungie, PA, USA). The temperature was monitored with a silicon diode sensor connected to a digital temperature controller (Scientific Instruments, model 9650–1, West Palm Beach, FL, USA). Infrared spectra were collected after cooling the sample to 10 K. Spectra were recorded in transmission mode using a Bruker IFS 66 Fourier transform infrared spectrometer (Bruker Optik, Ettlingen, Germany) equipped with a liquid-nitrogen-cooled MCT detector. Each spectrum was averaged over 128 scans at a spectral resolution of 0.5 cm^–1^, in the 4000–450 cm^–1^ spectral range.

### Computational Details

All computations have been performed using the Gaussian16 (revision C.02) suite of programs.^55^ Equilibrium structures and anharmonic force fields of FA and its 1:1 FAW complexes have been determined with very-tight convergence criteria by employing the global hybrid B3LYP^56^ and the double-hybrid rev-DSD-PBEP86 ^57–59^ density functionals. Both DFT models incorporate Grimme’s dispersion^60^ D3 correction^61^ with Becke–Johnson (BJ) damping function.^62^ They are shortly denoted as B3D3 and revDSD, respectively, in the following. Computations have been performed in conjunction with the “seasonal” jun-cc-pVTZ^63^ basis set (abbreviated in the following as junTZ). These combinations of functionals and basis set proved to be efficient in obtaining reliable structural parameters and for the calculation of vibrational properties at the anharmonic level.^64,65^ For the FAW complexes, calculations have been extended to incorporate the counterpoise correction (CP), to be compared with those not accounting for it (NCP).^66,67^

The FAW complexes considered in this work are three, which are those previously observed by means of microwave spectroscopy by Blanco *et al.* in ref 43. They are indicated as FAW-Ia, FAW-Ib, and FAW-Ic; their structures are shown in Figure 1 together with the atom labeling.

**Figure 1:**
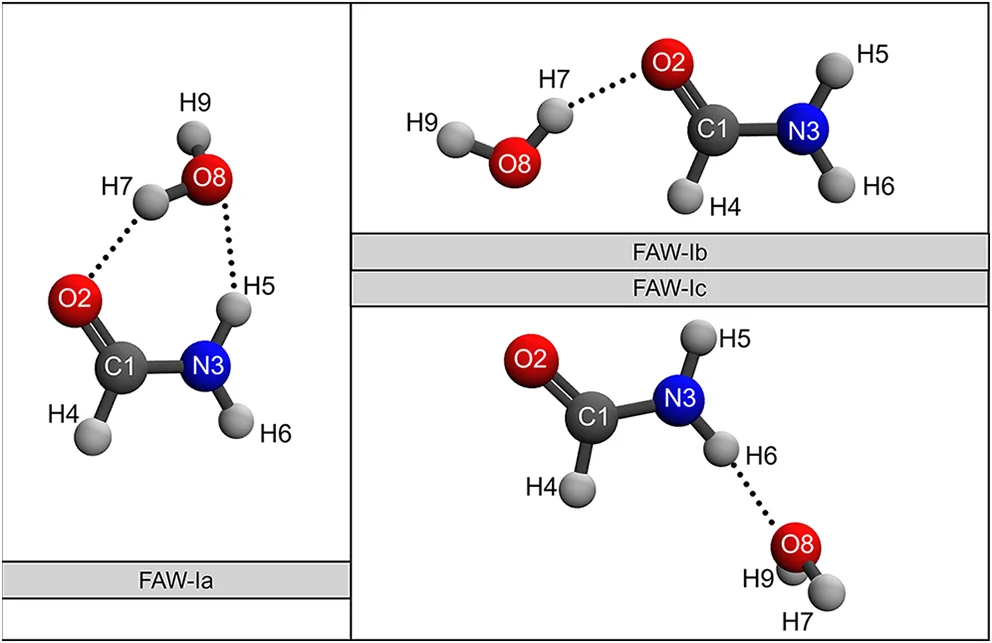
Three complexes of formamide and water (1:1) considered in this work together with the atom labeling.

VPT2 was applied to anharmonic force fields obtained by numerical differentiation of analytical Hessians, as implemented in Gaussian16 and described in detail in ref 68. Here, we employed the generalized version of VPT2 (GVPT2)^68–71^ in combination with the reduced dimensionality (RD) model.^72,73^ This choice is meant to remove large-amplitude motions (LAMs) and low vibrational frequencies of FAW complexes from the perturbation treatment. In this work, all force constants involving normal modes lying below 500 cm^–1^ were excluded from the VPT2 treatment.^74,75^ Additionally, for FAW complexes, hybrid anharmonic potential energy (PES) and corresponding spectroscopic property (PS) surfaces have also been built following the ”PES + PS” procedure described in ref 76. In detail, the harmonic terms, computed at the higher level of theory (here revDSD), are combined with the anharmonic ones, computed at a lower level (B3D3), in order to create hybrid PES and PS expressed as a Taylor series in function of dimensionless normal coordinates. This approach requires that the normal coordinates at the two levels of theory are identical, which is automatically checked by computing the normal mode overlap, as proposed by Duschinsky.^77^ This hybrid model is denoted as revDSD/B3D3 in the following.

## Results and Discussion

### Structure and Spectroscopic Parameters of Formamide

The formamide structural and spectroscopic parameters computed at different levels of theory are compared in Tables 1 and 2, respectively, with the reference experimental data being also reported.

**Table 1:** Structural Parameters and Ground State Rotational Constants^a^ of Formamide at Different Levels of Theory^b^

parameter^c^	B3D3		revDSD	CCSD(T)/CBS+CV	*r*_e_^SE^
C1O2	1.2108		1.2144	1.2097	1.212(2)
C1N3	1.3567		1.3582	1.3543	1.354(2)
H4C1	1.1037		1.1028	1.0997	1.097(3)
N3H5	1.0067		1.0061	1.0034	1.008(2)
N3H6	1.0040		1.0035	1.0007	1.017(2)
O2C1N3	124.86		124.73	124.58	124.2(5)
H4C1O2	122.68		122.71	122.70	123.4(5)
H5N3C1	119.49		119.37	119.18	119.9(1)
H6N3C1	121.25		121.11	121.08	120.5(2)
*A*_0_	72478.90 [−0.33]^d^		72158.68[−0.77]	72620.35 [−0.13]	71468.16 [−1.72]
*B*_0_	11307.34 [−0.58]		11280.84 [−0.82]	11355.33 [−0.16]	11386.59 [0.11]
*C*_0_	9797.91 [−0.37]		9772.29 [−0.63]	9836.44 [0.03]	9838.82 [0.05]

a Equilibrium rotational constants augmented by vibrational corrections computed at the revDSD level (MHz). Distances and angles are expressed in Å and degrees, respectively.

b For comparison, the semi-experimental equilibrium (*r*_e_^SE^) and CCSD­(T)/CBS+CV structures are reported.^78^

c For atom labeling, see the structure of formamide in Figure 1.

d Relative deviations (%) from the experimental rotational constants^43^ (*A*_0_ = 72716.8847(62) MHz, *B*_0_ = 11373.5814(11) MHz, *C*_0_ = 9833.8812(11) MHz) are reported in square brackets.

**Table 2:** Fundamental Vibrational Wavenumbers^a^ of Formamide at Different Levels of Theory, compared with Selected Experimental Data. Wavenumbers in cm^–1^, IR Intensities in km/mol

Assignment		B3D3						revDSD						Exp.			Mode description
		Harm.		NRD		RD		Harm.		NRD		RD		ref 86.	This Work		
		ω	IR	ν	IR	ν	IR	ω	IR	ν	IR	ν	IR	Gas	Ar	Abs.^b^	
A’	ν_1_	3716	43	3524	32	3542	37	3754	50	3550	36	3587	46	3563.7	3547	0.09	asym. NH_2_ str.
	ν_2_	3578	34	3409	16	3427	21	3609	45	3431	26	3460	38	3439.5	3427	0.12	sym. NH_2_ str.
	ν_3_	2943	91	2718	40	2717	40	2992	83	2867	65	2866	67	2853.2	2883	0.12	CH str.
	ν_4_	1786	433	1754	384	1751	406	1788	425	1756	362	1752	400	1754.2	1738	0.43	C=O str.
	ν_5_	1618	59	1576	45	1568	48	1626	52	1585	37	1577	40	1578.2	1576	0.12	in plane NH_2_ sci.
	ν_6_	1418	6	1389	4	1388	5	1422	6	1395	4	1393	5	1390.7	1396	0.03	in plane CH sci.
	ν_7_	1266	112	1248	60	1250	84	1278	115	1256	99	1259	97	1258.1	1262	0.08	CN str.
	ν_8_	1056	5	1050	10	1034	5	1058	5	1056	11	1038	6	1033.3	1046	0.02	in phase NCO/NH_2_ bend
	ν_9_	569	11	569	6	566	10	567	11	569	7	565	10	565.83	565	0.04	out of phase NCO/NH_2_ bend
A”	ν_10_	1042	3	1019	2	1021	2	1049	4	1024	2	1028	3	1033.3	1034	0.06	out of planeCH def.
	ν_11_	639	15	605	23	603	15	638	13	585	23	600	13	602.39	606	0.05	torsion
	ν_12_	245	204	448	88		204	159	211	714	737		211	288.98	303^c^		out of plane NH_2_
MAE^d^	Gas	51		23		19		64		9		7			9		
	Ar	50		24		22		64		9		12					

a Harmonic and anharmonic wavenumbers are denoted as ω and ν, respectively.

b Experimental IR intensities reported as absorbance at band maximum.

c From MI-FTIR spectra, ref 50.

d MAE with respect to the experimental data; mode ν_12_ is excluded from the statistics.

A detailed analysis of the accuracy of the computed structural parameters is possible owing to the availability of a semiexperimental equilibrium structure (*r*_e_^SE^) and the CCSD­(T)/CBS+CV equilibrium geometry, both determined in ref 78. The latter structure exploits the CCSD­(T) method (coupled-cluster with all single and double excitations and a quasiperturbative treatment of triples)^79^ in combination with the extrapolation to the complete basis-set (CBS) limit and accounting for core–valence (CV) correlation effects. According to the literature,^80–82^ the CCSD­(T)/CBS+CV level of theory is expected to provide bond distances and angles with an accuracy of 1–2 mÅ and 0.05–0.1°, respectively. Instead, *r*_e_^SE^ is an equilibrium geometry of experimental quality, which is obtained from the fit of the semiexperimental equilibrium rotational constants (*B*_e_^SE^) for different isotopic species to the geometrical parameters.^83^ In turn, the *B*_e_^SE^ values are derived by correcting the experimental ground-state rotational constants with computed vibrational corrections. Therefore, the accuracy of the *r*_e_^SE^ structure strongly depends on the availability of a sufficient number of experimental data (for a balanced fit).^81,84^

The comparison with the reference data (Table 1) points out that the B3D3 and revDSD structures have a good accuracy. Indeed, the mean absolute error (MAE) with respect to both the *r*_e_^SE^ and CCSD­(T)/CBS+CV references is 3–6 mÅ and 0.1–0.6° for bond lengths and valence angles, respectively. The revDSD structure overestimates the C=O, C–N and C–H bond lengths, while the B3D3 similarly over estimates C–N and C–H but yields a C=O bond shorter than the *r*_e_^SE^ one. In detail, both revDSD and B3D3 predict the C=O bond distance within 1–2 mÅ from the reference *r*_e_^SE^ structure, while larger differences of 3–4 mÅ are observed for the C–N one. Noted is that the CCSD­(T)/CBS+CV structural parameters agree with the *r*_e_^SE^ ones within the expected uncertainties, the only exception being the N3H6 bond length (for atom labeling see Figure 1). Interestingly, B3D3 and revDSD provide a N3H5 distance which is in agreement with *r*_e_^SE^, but only slightly underestimated. Instead, a relevant discrepancy (∼13 mÅ) is noted for N3H6, which seems to be wrongly determined by the semiexperimental approach. Indeed, the *r*_e_^SE^ value of 1.017 Å is 16 mÅ longer than that at the CCSD­(T)/CBS+CV level. This discrepancy clearly points out the difficulty of describing the NH_2_ moiety by means of the semiexperimental methodology because of the LAM. Therefore, the value of 1.001 Å determined at the CCSD­(T)/CBS+CV level is the best prediction of the N3H6 distance, with the revDSD and B3D3 levels only slightly overestimating it by ∼3 mÅ.

Table 1 also collects the rotational constants corresponding to the structures there reported. In all cases, the equilibrium rotational structures, straightforwardly derived from the equilibrium geometry,^81,85^ are augmented by vibrational corrections at the revDSD level. If one compares these computed rotational constants with the experimental values,^43^ it is observed that B3D3 performs slightly better, in terms of MAE, with respect to revDSD. The CCSD­(T)/CBS+CV level provides the smallest MAE, i.e., 0.11%, while *r*_e_^SE^ reproduces well the *B*_0_ and *C*_0_ rotational constants. Instead, *A*_0_ (not considered in the fitting procedure to determine *r*_e_^SE^) is wrongly predicted, the corresponding deviation being 1.72%.

Moving to the vibrational properties reported in Table 2, we start by noting that the harmonic wavenumbers of the NH_2_ and C–H stretching modes computed at the B3D3 level are lower than those from revDSD by about 30–50 cm^–1^, while a good agreement between the two levels is observed for the C=O stretching. A note is deserved for the ν_12_ mode, i.e., the NH_2_ out-of-plane vibration. This low-energy LAM is poorly determined at the harmonic level because of the limitations of the approximation, and is even worse described by VPT2 due to the inadequacy of perturbation theory.^87^ Furthermore, this mode shows the greatest discrepancy, about 85 cm^–1^, between the B3D3 and revDSD harmonic wavenumbers, with the B3D3 value being the closest to the experiment (deviation of about 40 cm^–1^). Interestingly, such a discrepancy is not observed for the harmonic IR intensity, with an agreement between B3D3 and revDSD within 2%. However, at both levels, anharmonic NRD computations yield excessive correction to intensity, thus highlighting the inadequacy of perturbative treatments for ν_12_. As mentioned in the computational section, this vibrational mode and all related force constants were excluded from the GVPT2 treatment within the RD scheme. The comparison of the results issuing from the RD scheme with the standard GVPT2 ones (NRD values) points out significant differences in those modes involving the NH_2_ group, with the largest deviation being about 20–40 cm^–1^ for the NH_2_ stretching vibrations. Instead, the remaining fundamental bands are nearly unaffected, with differences as small as 1–4 cm^–1^.

Considering the gas-phase experiment as reference and excluding the NH_2_ LAM from the statistics, the NRD-B3D3 wavenumbers show a MAE of about 1.1% (23 cm^–1^), while the RD scheme reduces it to 0.8% (19 cm^–1^). Focusing on revDSD, the NRD MAE is 0.8% (9 cm^–1^), which becomes as small as 0.3% (7 cm^–1^) once the RD scheme is applied. Notably, in the case of B3D3, the largest discrepancy with respect to experiment (136–166 cm^–1^) is noted for the ν_3_ band, which is the C–H stretching and is thus not affect by the RD scheme. This band, predicted at 2717 cm^–1^ by B3D3, has to be compared with the gas-phase value of 2853 cm^–1^ and with 2883 cm^–1^ in the case of MI-FTIR.

The comparison between gas-phase and low-temperature Ar-matrix experimental results points out a red-shift of 16 cm^–1^ for the characteristic and intense C=O stretching band. A similar shift is also observed for the symmetric and asymmetric NH_2_ stretching modes, which are observed in matrix at 3427 and 3547 cm^–1^, respectively, to be compared with 3439.5 and 3563.7 cm^–1^ in the gas phase. Such matrix shifts, that are commonly observed,^88,89^ indicate that both NH_2_ and C=O groups are affected by the interaction with the matrix. Overall, the fundamental wavenumbers in the spectrum measured in Ar-matrix exhibit relative deviations, with respect to gas-phase experiments, that are well within 0.5%, with the A″ symmetry modes showing the smallest differences.

In view of the comparison addressed above, in the discussion on IR spectra that follows, only RD-GVPT2 computations are considered. The detailed analysis of the characteristic vibrational features of formamide in the matrix environment indicates an agreement with MI-FTIR data of 19 cm^–1^, on average, for B3D3, and 12 cm^–1^ for revDSD. These larger discrepancies are somewhat expected considering that revDSD well describes gas-phase data and that MI-FTIR data are shifted as discussed above. For the C=O stretching, both B3D3 and revDSD show a blue-shift of about 15 cm^–1^ with respect to the MI-FTIR value of 1738 cm^–1^. Overall, these results suggest that some systematic corrections for the Ar-matrix environment might be feasible.^90^ Furthermore, taking the MI-FTIR data from this work as reference, we note that all computations at the RD-GVPT2 level correctly reproduce relative IR intensities for all fundamental bands.

The comparison between theory and experimental data from this work enabled us to confirm prior assignments^50,86^ as well as to extend the spectroscopic characterization to additional features, as indicated in Figure 2. First, we confirm the assignment for the features observed in the 700–450 cm^–1^ range that show comparable intensities: at 565 cm^–1^ lies the ν_9_ fundamental band, at 606 cm^–1^ the ν_11_ one and at 678 cm^–1^ the 2ν_12_ overtone. The peaks associated with the NH_2_-group vibrational modes show a particular shape, probably due to site splitting arising from different trapping sites in the Ar matrix. This characteristic multiplet structure, combined with the positions of combination bands and overtones estimated using the experimental fundamental wavenumbers, allows us to extend the spectral assignments to features involving ν_12_ that lie outside the RD-GVPT2 treatment. In particular, we have assigned the peak at 887 cm^–1^ to the ν_9_ + ν_12_ combination. For the cluster of weak bands around 1200–1150 cm^–1^, only the most intense feature at 1194 cm^–1^ could be firmly assigned to the 2ν_11_ overtone, with the GVPT2 wavenumber being 1178 cm^–1^ and the corresponding IR intensity 23.4 km/mol. At 1183 cm^–1^ and 1206 cm^–1^, two signals are observed that might be combination modes involving the NH_2_ wagging: ν_9_ + 2ν_12_ and ν_11_ + 2ν_12_. The feature at 2766 cm^–1^ has been assigned to 2ν_6_ (2745 cm^–1^, 23.8 km/mol). Other peaks have been observed in the spectra but, due to their extremely low intensity, they have not been further considered. These weak unassigned features may also originate from overtone and combination bands involving low-frequency mode excluded from the perturbative treatment.

**Figure 2:**
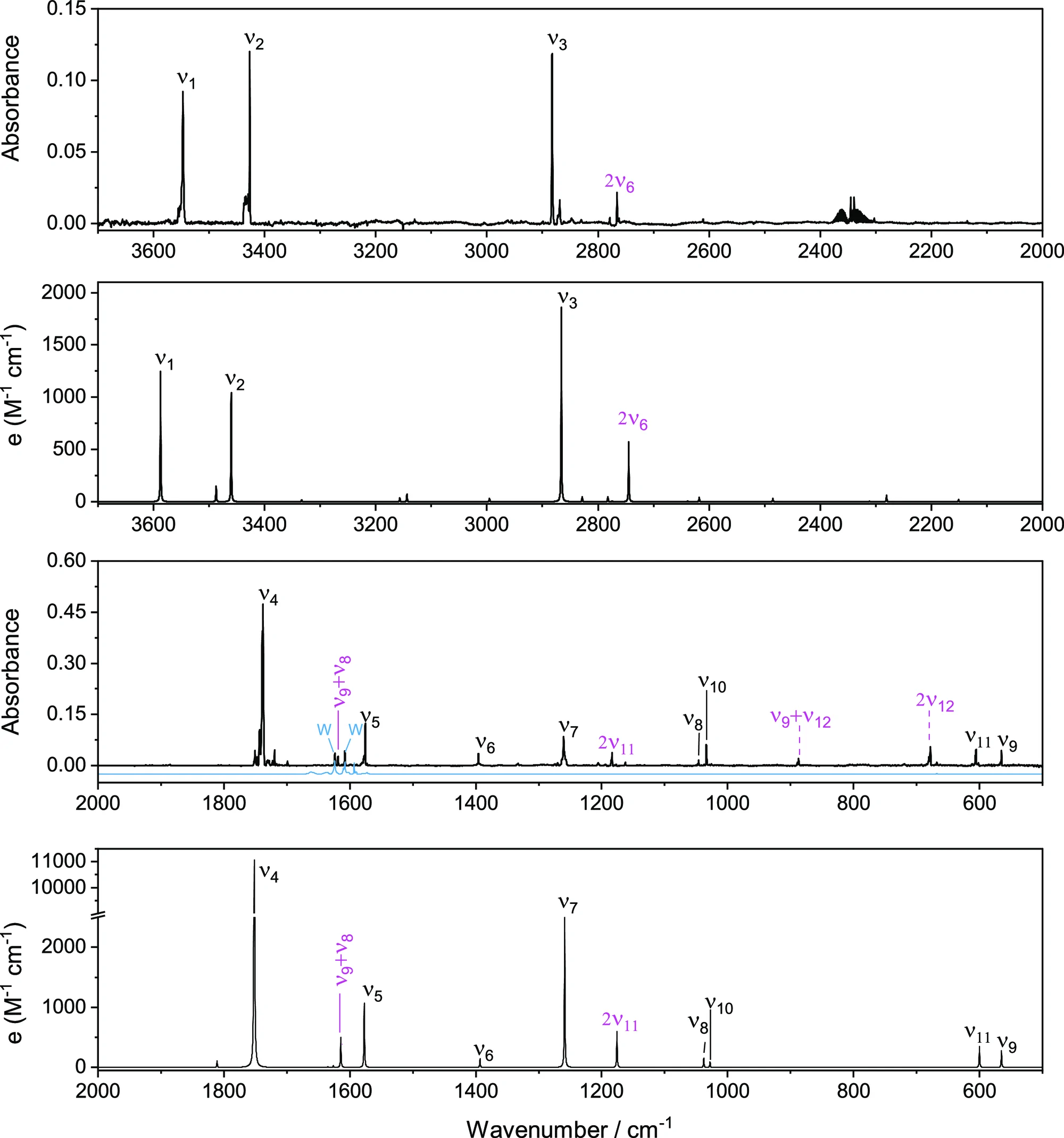
IR spectrum of the formamide (FA) monomer in an Ar matrix reported in two ranges: 3700–2000 cm^–1^ and 2000–500 cm^–1^. For each region, the upper panel shows the experimental spectrum, while the bottom one reports the fully anharmonic RD-GVPT2 spectrum computed at the revDSD level. The assignment for the most intense bands is also provided. Experimental absorption features arising from residual water trapped in the matrix are shown in cyan and labeled as W. New assignments are highlighted in magenta, while tentative assignments are indicated by dashed lines. The theoretical stick spectra were convoluted with Gaussian profiles using a half-width at half-maximum (HWHM) of 1 cm^–1^.

The complete assignment of the most characteristic FA bands shown in Figure 2 opens the way to the investigation of how the IR spectrum changes upon interaction of formamide with water molecules. This will be analyzed considering binding patterns previously observed either in Ar matrices^34,47^ or in the gas phase.^43^

### Formamide and Its Water Complexes: Structure and IR Spectra

Some key structural parameters of the FAW-Ia, FAW-Ib, and FAW-Ic complexes (see Figure 1), computed at the B3D3 and revDSD levels, are collected in Table 3 together with the corresponding rotational constants. The complete set of structural parameters is listed in Tables S1–S3 of the Supporting Information (SI).

**Table 3:** Key Structural Parameters of the FAW-Ia, FAW-Ib, and FAW-Ic Formamide-Water Complexes at the B3D3 and revDSD Levels Together with the Corresponding Rotational Constants^a^

		B3D3			revDSD	
la	parameter	CP	NCP		CP	NCP
	C1O2	1.2225	1.2226		1.2247	1.2250
	O8H7	0.9776	0.9778		0.9744	0.9753
	O8H9	0.9604	0.9604		0.9599	0.9600
	N3H5	1.0145	1.0146		1.0130	1.0133
	N3H6	1.0036	1.0036		1.0035	1.0035
	O2···H7	1.8786	1.8756		1.9124	1.8917
	O8···H5	2.0616	2.0592		2.0773	2.0612
	N3···O8	2.8859	2.8835		2.9028	2.8860
	O2···O8	2.7770	2.7746		2.8028	2.7863
	C1O2H7	107.40	107.37		107.45	107.19
	O2H7O8	151.47	151.53		150.67	151.22
	O8H5N3	136.79	136.77		137.14	136.97
	*A*_0_	11186.04 [−0.37]^b^	11185.93 [−0.37]		11146.24 [−0.73]	11188.46 [−0.35]
	*B*_0_	4562.24 [−0.54]	4570.82 [−0.35]		4489.69 [−2.12]	4579.16 [−0.17]
	*C*_0_	3244.53 [−0.44]	3249.02 [−0.30]		3203.49 [−1.70]	3238.19 [−0.63]

		B3D3			revDSD	
lb	parameter	CP	NCP		CP	NCP
	C1O2	1.2185	1.2186		1.2215	1.2217
	O8H7	0.9737	0.9739		0.9713	0.9719
	O8H9	0.9602	0.9602		0.9598	0.9597
	N3H5	1.0069	1.0069		1.0063	1.0064
	N3H6	1.0040	1.0040		1.0037	1.0037
	O2···H7	1.8811	1.8781		1.9179	1.8993
	O2···O8	2.8097	2.8066		2.8289	2.8122
	C1O2H7	103.62	103.55		101.03	100.74
	O2H7O8	158.44	158.41		155.17	155.40
	*A*_0_	28659.46 [9.51]^b^	28616.32 [9.34]		26421.26 [0.96]	26346.56 [0.67]
	*B*_0_	2602.37 [−3.00]	2607.89 [−2.80]		2660.60 [−0.83]	2688.02 [0.19]
	*C*_0_	2374.85 [−2.42]	2379.26 [−2.84]		2403.07 [−1.26]	2424.90 [−0.37]

		B3D3			revDSD	
lc	parameter	CP	NCP		CP	NCP
	C1O2	1.2145	1.2145		1.2178	1.2178
	O8H7	0.9618	0.9619		0.9611	0.9612
	N3H5	1.0065	1.0065		1.0061	1.0061
	N3H6	1.0101	1.0101		1.0089	1.0091
	O8···H6	2.0065	2.0030		2.0326	2.0140
	O8···N3	3.0154	3.0119		3.0394	3.0213
	O8H6N3	176.62	176.61		175.50	175.79
	H6O8H7	124.76	125.14		126.35	125.90
	*A*_0_	34558.37	34631.26		33907.24	34055.05
	*B*_0_	2072.65 [−0.58]^b^	2074.71 [−0.48]		2057.79 [−1.29]	2071.78 [−0.62]
	*C*_0_	1968.92 [0.98]	1971.01 [−1.09]		1953.24 [0.18]	1966.42 [0.85]

a Distances and angles are in Å and degrees, respectively. Equilibrium rotational constants augmented by vibrational corrections at the NCP-revDSD level from NRD-GVPT2 calculations (MHz).

b Relative deviations (%) with respect to the experimental rotational constants from ref 43 are reported in square brackets. For FAW-Ia, the experimental rotational constants are *A*_0_ = 11227.9330(23) MHz, *B*_0_ = 4586.9623(16) MHz, *C*_0_ = 3258.8277(12) MHz. For FAW-Ib, the experimental rotational constants are *A*_0_ = 26170.8(61) MHz, *B*_0_ = 2682.97653(65) MHz, *C*_0_ = 2433.84992(64) MHz. For FAW-Ic, the experimental values of the 0 sublevel are *B*_0_ = 2084.6937(141) MHz, *C*_0_ = 1949.7643(136) MHz. For FAW-Ic, *A*_0_ was not determined experimentally, thus is not considered in the statistics.

From the inspection of Table 3, it is noted that both NCP and CP computations yield qualitatively equivalent structures. To gain insights into their accuracy, the corresponding rotational constants are compared with their experimental counterparts. To perform such comparison, as done for formamide, the equilibrium rotational constants (straightforwardly derived from the equilibrium geometries) are augmented by revDSD vibrational corrections. The data in Table 3 suggest that, at the B3D3 level of theory, the NCP and CP geometries behave on average similarly, but can show deviations as large as 9% (FAW-Ib, *A*_0_) with respect to experiment. This large discrepancy might be due to the out-of-plane position of the H9 atom, i.e., the H atom of water not involved in the hydrogen bond. Indeed, the H9O8H7O2 dihedral angle has a value of 156° at the B3D3 level for both CP and NCP structures, while the revDSD predicts the same dihedral angle to be 162°.

For the FAW-Ia complex, the NCP-B3D3 and NCP-revDSD rotational constants show the smallest relative MAEs: 0.34% and 0.37%, respectively. While the CP correction does not seem to play a relevant role for B3D3, a different trend is observed for revDSD. Considering the three complexes, the NCP-revDSD structures lead to rotational constants that show smaller deviations from experiment than the CP counterparts. Overall, the comparison between computed and experimental rotational constants points out that CP-/NCP-B3D3 predicts rotational constants with an error of about 2% and CP-revDSD with an error of 1.1%. The best performance is obtained from NCP-revDSD, with a global MAE of 0.48%. This result is in line with MAE values of 0.4–0.5% observed for other molecular species, such as polycyclic aromatic hydrocarbons and protonated species.^91,92^ This suggests that the performance of revDSD is independent of the system under investigation and also extends to molecular complexes, thus making the revDSD level a solid reference. The present results support the use of NCP-revDSD as the level of choice also for larger complexes and molecular adducts.

The similarity of CP and NCP structures at the B3D3 level is made evident by the comparison of the corresponding rotational constants, and it is well reflected in the structural parameters. Across the three complexes, distances agree within 1 mÅ for all intramolecular bonds, and within up to 2–4 mÅ for the intermolecular ones. Similarly, the angles agree within 0.5° or less. Focusing on CP and NCP results at the revDSD level, the error on the rotational constants points out that the NCP computations provide a better structure than the CP ones. The differences are indeed noticeable, especially for intermolecular parameters. Indeed, intramolecular bond lengths agree within 1 mÅ, while the distances describing the interactions between the two fragments show deviations in the order of 15–20 mÅ, with the CP values being always longer than NCP ones. Overall, B3D3 (either CP or NCP) yields the shortest intermolecular distances and CP-revDSD the longest ones, with the NCP-revDSD values lying in between. These deviations span a range of 20–40 mÅ for distances involving the H atom, while they are up to 30 mÅ for those including only heavy atoms.

For FAW-Ia, our intermolecular parameters can be compared with those of the partial semiexperimental equilibrium structures derived in ref 93. According to them, the O8–H5 distance is 2.150(2) Å, and thus about 90 mÅ longer than the NCP-B3D3 and NCP-revDSD values. For the O8H5N3 angle, we note a semiquantitative agreement; this angle is predicted to be 132.55(5)° by the semiexperimental approach,^93^ while it has been found to be 136–137° by the present computed structures. While the different partial semiexperimental equilibrium structures of ref 93 provide O8–H5 distances that differ one from the other up to 50 mÅ, the CP-B2PLYP-D3BJ/jun-cc-pVTZ level—also employed in ref 93—leads to a distance of 2.0573 Å, which is consistent with those derived in this work. The comparison addressed above suggests that partial semiexperimental equilibrium structures should be taken with caution because can be affected by flaws related to the lack of experimental data.

All computations consistently predict the structures of the FAW complexes and the changes in intramolecular parameters with respect to the isolated formamide due to the interaction with water. These changes clearly reflect different bonding patterns. Namely, the C1O2 distance becomes longer in all complexes, but the magnitude of this modification varies from ∼10 mÅ for FAW-Ia, where C1O2 is involved in a cyclic structure, to 7 mÅ for FAW-Ib, which is stabilized by the O2···H7–O8 HB, and to only 3 mÅ for FAW-Ic, where O2 is not involved in any direct interaction with water. For the latter complex, the interaction mainly involves the NH_2_ group, as indicated by the increase of the N3H6 bond length by about 6 mÅ with respect to the isolated FA. Similarly, we observe the lengthening of the N3H5 bond distance by 7 mÅ in the cyclic FAW-Ia structure. Focusing on the C1N3 bond, in all FAW complexes, this is shorter than that of formamide. The largest deviations are shown by FAW-Ia, where two HBs, namely O2···H7–O8 and N3–H5···O8, are present.

Moving to the analysis of IR spectra, we first note that the FAW-Ia and FAW-Ic complexes were previously observed in the MI-FTIR by Engdahl *et al.* ^47^ and the presence of FAW-Ib was also postulated. However, Engdahl *et al.* were not able to find any feature of a FAW complex having small NH_2_ stretching shifts, as expected for a complex solely involving an O···H–O HB. In ref 47, the lack of FAW-Ib was explained by invoking the possible rearrangement to the most stable cyclic FAW-Ia form during the preparation of the matrix. However, FAW-Ib was observed in the gas phase in FT-MW experiments,^43^ this explaining the reason why we have considered it in this work. To provide an accessible evidence of the FAW features to the reader, the experimental MI-FTIR spectra of the FA/H_2_O/Ar mixture have been combined with those of the two monomers (FA/Ar and H_2_O/Ar), which were recorded in the same conditions. In the resulting difference spectrum (black trace in Figure 3), positive bands are due to features that are absent in the monomer spectra, while negative ones correspond to those of the monomers and their aggregates.^49,94^ Additional conformer-specific information is provided by the annealed-minus-deposited difference spectrum (gray trace in Figure 3), obtained by subtracting the spectrum of the deposited sample from that recorded after annealing at 30 K for 5 min: positive features indicate bands that gained intensity upon annealing, while negative ones those that decreased their intensity. These difference spectra are complemented by theoretical predictions, at the RD-GVPT2 NCP-revDSD level, for all FAW complexes and both monomers. A numerical form of these spectra is also provided in the Supporting Information (DAT files), while Tables 4 and S4–S6 report the harmonic and anharmonic wavenumbers and IR intensities obtained from CP and NCP calculations at the B3D3 and revDSD levels.

**Table 4:** Key Fundamental Wavenumbers^a^ for the Formamide-Water FAW-Ia, FAW-Ib, and FAW-Ic Complexes Computed at the B3D3, revDSD and Hybrid Levels, Compared with the Experimental MI-FTIR Data^b^

		B3D3				revDSD					revDSD/B3D3	Exp.
		CP		NCP		CP		NCP			NCP	(this work)
la	assignment^c^	ω	ν	ω	ν	ω	ν	ω	νNRD	ν	ν	
	ν_3_(W)	3877	3692	3876	3692	3905	3717	3903	3734	3717	3727	3706
	ν_1_(W)	3541	3356	3538	3353	3615	3414	3599	3441	3396	3419	3430
	ν_1_(FA)	3691	3521	3691	3521	3727	3556	3726	3553	3556	3563	3532
	ν_2_(FA)	3472	3276	3471	3273	3523	3356	3517	3366	3347	3330	3355
	ν_4_(FA)	1756	1718	1756	1717	1768	1728	1767	1734	1727	1729	1717
	MAE^d^	120	36	118	37	160	13	154	17	17	20	
	|MAX|^e^	171	79	170	82	199	24	197	28	35	31	

		B3D3				revDSD					revDSD/B3D3	Exp.
		CP		NCP		CP		NCP			NCP	(this work)
lb	assignment^c^	ω	ν	ω	ν	ω	ν	ω	νNRD	ν	ν	
	ν_3_(W)	3880	3704	3879	3704	3909	3729	3908	3734	3729	3741	3715
	ν_1_(W)	3603	3402	3600	3398	3666	3481	3654	3512	3474	3485	3514
	ν_1_(FA)	3715	3536	3715	3536	3752	3576	3752	3567	3576	3580	3555
	ν_2_(FA)	3578	3436	3578	3437	3609	3456	3608	3444	3452	3447	3465
	ν_4_(FA)	1763	1726	1762	1726	1770	1734	1770	1743	1733	1735	1725
	MAE^d^	113	34	112	35	146	17	144	14	19	22	
	|MAX|^e^	165	112	164	116	197	33	197	21	40	29	

		B3D3				revDSD					revDSD/B3D3	Exp.
		CP		NCP		CP		NCP			NCP	(this work)
lc	assignment^c^	ω	ν	ω	ν	ω	ν	ω	νNRD	ν	ν	
	ν_3_(W)	3905	3724	3905	3724	3938	3755	3937	3751	3752	3762	3726
	ν_1_(W)	3804	3651	3804	3650	3826	3670	3824	3651	3669	3676	3640
	ν_1_(FA)	3671	3488	3671	3488	3714	3534	3712	3543	3533	3532	3524
	ν_2_(FA)	3518	3357	3518	3357	3561	3408	3557	3417	3404	3397	3389
	ν_4_(FA)	1776	1740	1776	1740	1780	1744	1780	1742	1741	1744	1731
	MAE^d^	133	18	133	17	162	20	160	19	18	20	
	|MAX|^e^	179	36	179	36	212	30	211	28	29	36	
	MAE ALL^f^	122	29	121	30	156	17	153	17	18	21	
	|MAX|ALL^g^	179	112	179	116	212	33	211	28	40	36	

a Harmonic and anharmonic wavenumbers are denoted as ω and ν, respectively. Anharmonic computations are at the RD-GVPT2 level, except those denoted as νNRD.

b All data in cm^–1^.

c Intramolecular vibrations of formamide and water monomers within the FAW complex are labeled as (FA) and (W), respectively.

d MAE with respect to the experimental data.

e Maximum absolute error with respect to the experimental data.

f MAE with respect to the experimental data considering all FAW conformers.

g Maximum absolute error with respect to the experimental data considering all FAW conformers.

**Figure 3:**
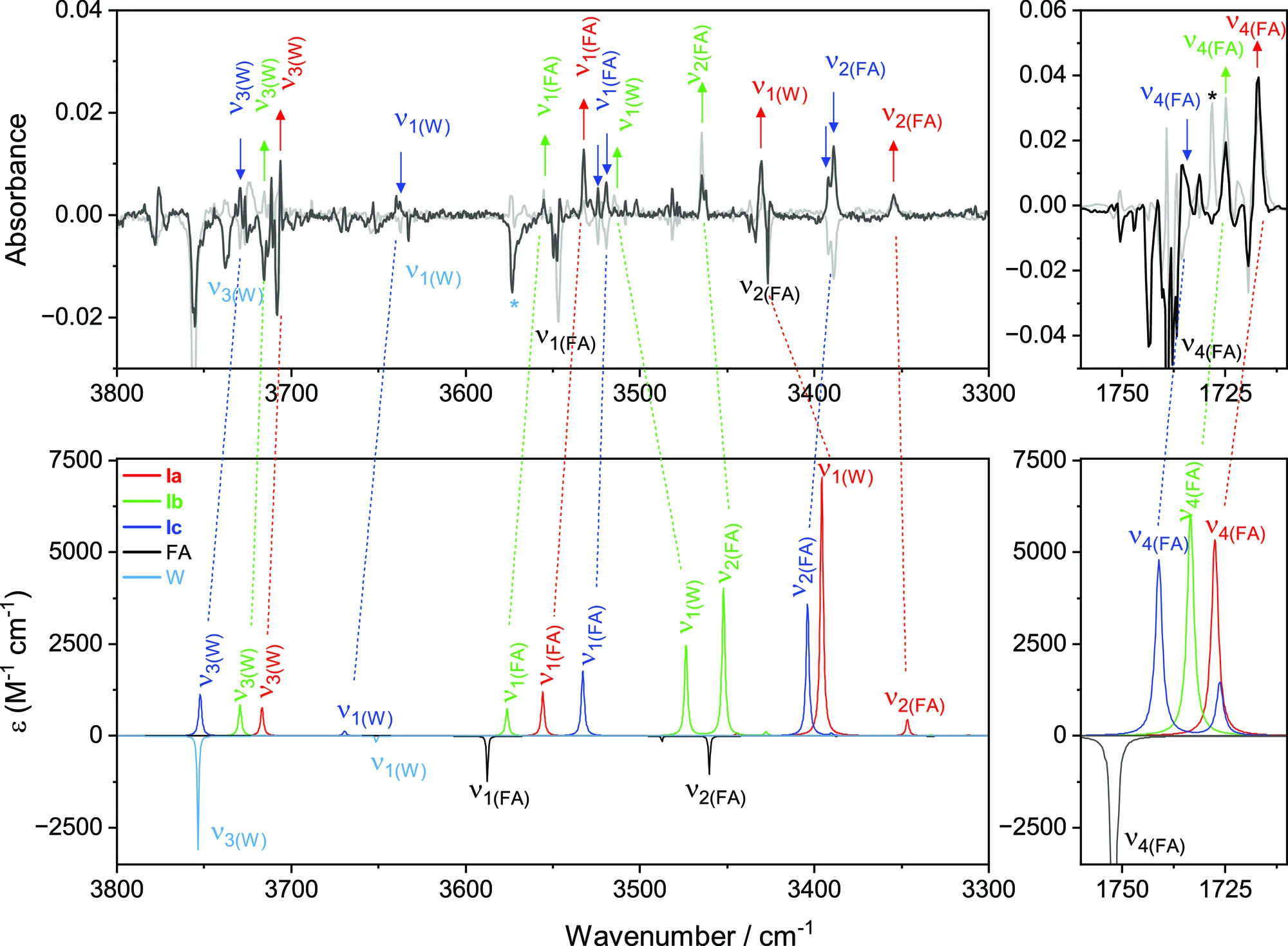
Top: OH/NH– and C=O–stretching regions of FTIR difference spectra of the FA/H_2_O/Ar system. The black trace was obtained by subtracting the spectra of isolated monomers, FA/Ar and H_2_O/Ar (1:1000 in Ar), from that of the FA/H_2_O/Ar mixture recorded at 10 K after deposition at 15 K. The gray trace corresponds to the difference spectrum obtained by subtracting the deposited sample spectrum from that recorded at 10 K after subsequent annealing at 30 K for 5 min. Positive features in the deposited-minus-monomer spectrum (black trace) correspond to bands of the formamide–water (FAW) complexes, while negative features correspond to isolated monomers and their aggregates, marked by an asterisk (blue for water, black for formamide). Band assignments are indicated by labels: subscripts FA and W denote formamide and water vibrations, respectively, within the FAW complexes. In the annealed-minus-deposited spectrum (gray trace), positive and negative features indicate bands that increase and decrease in intensity upon annealing, respectively. Color-matched arrows highlight the evolution of individual FAW conformer bands, with upward and downward arrows denoting increasing and decreasing intensities upon annealing. Bottom: Fully anharmonic RD-GVPT2 spectra computed at the NCP-revDSD level. Bands of the FAW complexes are shown as positive features, whereas bands of the isolated monomers are shown as negative features. Theoretical stick spectra were convoluted with Gaussian profiles with a half-width at half-maximum (HWHM) of 1 cm^–1^.

The comparison of theoretical results indicates that, at the B3D3 level, the CP and NCP approaches produce essentially identical harmonic and anharmonic wavenumbers, with a maximum difference of 5 cm^–1^ and an agreement within 1 cm^–1^ for most of the fundamental transitions. This is consistent with the agreement observed for structural parameters. Moving from structures to IR features, the differences between the CP- and NCP-revDSD results exacerbate. In particular, for ν_1_(W) of FAW-Ia and FAW-Ib, CP leads to harmonic values greater than the NCP ones by 16 and 12 cm^–1^, respectively. Interestingly, CP and NCP anharmonic corrections for this mode agree within 4 cm^–1^, thus the resulting anharmonic wavenumbers show the same trend as harmonic ones. The comparison with experiment reveals mean deviations that are similar for the CP and NCP approaches at both the B3D3 and revDSD levels. Overall, CP-revDSD performs slightly better, but the differences with respect to NCP-revDSD are negligible. Infrared intensities appear to be more sensitive to the CP correction than the vibrational wavenumbers, with noticeable differences already evident at the B3D3 level, particularly for modes involved in hydrogen bonding. Unlike vibrational wavenumbers, experimental band intensities assigned to the FAW complexes cannot be determined quantitatively. Nevertheless, all computational approaches predict intensity patterns that are qualitatively consistent with experimental observations. Therefore, in view of the structural analysis discussed above, the NCP schemes can be preferred for larger multicomponent systems, where CP corrections would be unfeasible. NCP-revDSD has also been selected to compare RD-GVPT2 computations with the NRD counterparts. Tables S4–S6 clearly show that, as expected, the lowest-energy LAM modes cannot be reliably treated by VPT2, NRD-GVPT2 producing excessively large positive or negative corrections to both wavenumbers and IR intensities that lack any physical meaning. The higher-energy vibrations are also affected in NRD treatments, the extent depending on the coupling with the LAM modes. For instance, for ν_4_(FA) (C=O stretching), the NRD value is blue-shifted by about 7–10 cm^–1^ with respect to the RD one in FAW-Ia and FAW-Ib, both having a O···H–O HB, while the wavenumber is not affected in FAW-Ic, where C=O is not involved in any intermolecular vibration with water. Overall, RD-GVPT2 also shows a good agreement with the experiment, providing further evidence that the reduced-dimensionality, LAM-free GVPT2 models represent a reliable approach for systems where the full VPT2 treatment cannot be applied.^74,75^ We conclude this analysis by noting that the hybrid revDSD/B3D3 scheme yields MAEs of 20–22 cm^–1^ and maximum absolute deviations (|MAX|) of about 35 cm^–1^, thus performing nearly on par with revDSD but showing a marked improvement over B3D3. Indeed, this latter level of theory exhibits a MAE of about 30 cm^–1^ and a |MAX| greater than 100 cm^–1^. Therefore, based on the discussion above, the RD-GVPT2 NCP-revDSD data have been selected for the detailed analysis presented in the following.

The assignment of the experimental spectra is based on the appearance of bands that are shifted with respect to their monomer counterparts due to intermolecular interactions, as reported in Table 5. Additional information was obtained from the annealing experiments, which revealed three distinct intensity-change patterns, each attributable to a different FAW conformer: one set of bands decreases in intensity upon annealing, while two sets show increases of different magnitudes. Guided by these annealing patterns, the largest experimental shift (208 cm^–1^) is observed for the band at 3430 cm^–1^, whose intensity slightly increases after annealing, and which was already assigned to ν_1_(W) of the FAW-Ia complex in ref 47. This is in good agreement with the anharmonic computations that predict a shift of 255 cm^–1^ with respect to the water monomer. In this work, we have identified a second strongly shifted band in the ν_1_(W) region: the peak at 3514 cm^–1^, shifted by 124 cm^–1^ with respect to isolated water. This band shows a significant intensity increase upon annealing and is assigned to FAW-Ib, the conformer stabilized by an O–H­(W)···O­(FA) HB with a free NH_2_ group. This assignment is supported by the NCP-revDSD prediction of this transition at 3474 cm^–1^ (shift of 177 cm^–1^). This band lies very close to another previously unreported feature at 3465 cm^–1^, which agrees well with ν_2_(FA) of FAW-Ib, which is predicted at 3452 cm^–1^. Together, these two close-lying bands, ν_1_(W) and ν_2_(FA), show identical annealing behavior and form a characteristic pattern consistent with the simultaneous presence of an O–H­(W)···O­(FA) hydrogen bond and a free NH_2_ group, providing a confident assignment to FAW-Ib (see also Figure 3). It is worth noting that, for FAW-Ib, the computed shifts of ν_1_(FA) and ν_2_(FA) with respect to the formamide monomer are very small (−11 and −8 cm^–1^, respectively), reflecting the absence of any direct perturbation of the NH_2_ group in this conformer. The experimentally observed shifts for these modes are +8 and +38 cm^–1^, opposite in sign to the computed values. This apparent discrepancy may have both experimental and computational origins: the matrix-induced perturbation of the free NH_2_ vibrations may be different in the isolated monomer and in the FAW-Ib complex, thus introducing an uncertainty that cannot be captured by calculations on the corresponding isolated species. On the computational side, the near-degeneracy of ν_1_(W) and ν_2_(FA) gives rise to 1–1 Darling–Dennison resonance unique to FAW-Ib, which together with strong coupling between ν_1_(FA) and ν_2_(FA) result in a larger mode mixing.

**Table 5:** Intramolecular Vibrations of the Formamide and Water Monomers^a^ within the Formamide-Water FAW-Ia, FAW-Ib, and FAW-Ic Complexes^b^

		NCP-revDSD			Exp.	
	assignment^c^	ν	Δ	*I*	ν	Δ
ν_3_(W)	H_2_O	3753		56	3736	
	FAW-Ia	3717	–36	56	3706	–30
	FAW-Ib	3729	–24	61	3715	–21
	FAW-Ic	3752	–1	82	3726	–10
ν_1_(W)	H_2_O	3651		4	3638	
	FAW-Ia	3396	–255	511	3430	–208
	FAW-Ib	3474	–177	179	3514	–124
	FAW-Ic	3669	+18	10	3640	+2
ν_1_(FA)	FA	3587		46	3547	
	FAW-Ia	3556	–31	86	3532	–15
	FAW-Ib	3576	–11	53	3555	+8
	FAW-Ic	3533	–54	129	3524	–23
ν_2_(FA)	FA	3460		38	3427	
	FAW-Ia	3347	–113	32	3355	–73
	FAW-Ib	3452	–8	288	3465	+38
	FAW-Ic	3404	–56	260	3389	–38
ν_4_(FA)	FA	1752		400	1738	
	FAW-Ia	1727	–25	388	1717	–21
	FAW-Ib	1733	–19	439	1725	–13
	FAW-Ic	1741	–11	346	1731	–7

a Anharmonic wavenumbers are denoted as ν, while Δ refers to the shift with respect to monomer.

b RD-GVPT2 NCP-revDSD computations are compared with the experimental MI-FTIR Data (this work). wavenumbers incm^–1^, IR intensities (*I*) in km/mol.

c Intramolecular vibrations of the formamide and water monomers within the FAW complex are labeled as (FA) and (W), respectively.

Overall, we note a good agreement between experimental and computed shifts of the key bands, suggesting a reassignment of ν_1_(FA) and ν_2_(FA) with respect to the literature,^47^ as described in the following. In ref 47, the detailed analysis and bands assignment to FAW-Ia and FAW-Ic were based on experiments with different water isotopologues. Small but significant isotopic effects were observed for the most shifted band in the ν_1_(FA) region (3524 cm^–1^) and the least shifted band in the ν_2_(FA) region (3389 cm^–1^). In contrast, the least shifted ν_1_(FA) band at 3532 cm^–1^ and the most shifted ν_2_(FA) band at 3355 cm^–1^ remained unchanged regardless of the water isotopologue employed. The bands showing a clear isotopic effect were assigned to FAW-Ia. Our experiment indeed indicates the same grouping based on the annealing effects, with computations showing that NH stretching vibrations lose some symmetry with respect the isolated monomer, but still should be described as coupled vibrations despite complexation. Similar shifting patterns are found for ν_1_(FA)/ν_2_(FA) of FAW-Ia/Ic, but all levels of theory yield a larger shift for ν_1_(FA) and a smaller shift for ν_2_(FA) of FAW-Ic. For this reason, we propose a reassignment of these bands (with respect to ref 47), which is also supported by the fact that the largest shift of ν_2_(FA) is expected for FAW-Ia, where the N–H bond involved in cyclic hydrogen interaction is elongated by 7.2 mÅ, compared to 5.6 mÅ for FAW-Ic. Additionally, the significantly higher computed ν_2_(FA) IR intensity for FAW-Ic than for FAW-Ia (about 250 km/mol to be compared with less than 50 km/mol) is consistent with the greater experimental intensity of the band at 3389 cm^–1^ with respect to that at 3355 cm^–1^. We note, however, that the observed intensities also depend on the relative populations of the conformers trapped in the matrix.

Notably, we have been able to identify bands in the 3500–3450 cm^–1^ range that match well the close-lying ν_1_(W) and ν_2_(FA) predicted by the computations for FAW-Ib (see Figure 3), along with the remaining bands of this complex and additional features due to FAW-Ic, which were not reported in ref 47, as well as several reassignments for FAW-Ia. We were also able to assign additional signatures of all three complexes in the 1750–1250 cm^–1^ spectral range, as detailed in Tables 5 and S7. The three bands related to the C=O stretching, also shown in Figure 3, confirm that the most shifted one (1717 cm^–1^) belong to FAW-Ia,^47^ while those lying at 1725 and 1731 cm^–1^ refer instead to FAW-Ib and FAW-Ic, respectively.

Generally, the interaction-driven spectral features can be dissected with the aid of computations. Interaction with water leads to specific HBs: N–H­(FA)···O­(W) and/or O–H­(W)···O­(FA). The former induces elongation of the N–H bond and red-shifts of the NH_2_ stretching vibrations, the effect being most pronounced (50–100 cm^–1^) for the symmetric ν_2_(FA) mode. In contrast, the interaction of the C=O group with water determines a large red-shift (over 150 cm^–1^) of the water OH stretching modes. In detail, computations predict NH_2_(FA) red-shifts for FAW-Ia and FAW-Ic, and OH­(W) red-shifts for FAW-Ia and FAW-Ib, these outcomes being consistent with the cyclic, O–H­(W)···O­(FA) and N–H­(FA)···O­(W) structures of FAW-Ia, FAW-Ib and FAW-Ic, respectively. Interestingly, for all FAW complexes, the characteristic C=O stretching band is red-shifted with respect to the FA monomer, the shift magnitude ranging from about 10 cm^–1^ for FAW-Ic to more than 20 cm^–1^ for FAW-Ia. Notably, the computed shifts due to the hydrogen bonding agree well with the experimental observations in all cases.

## Conclusion

New IR spectra of formamide and formamide–water complexes have been measured in a low-temperature Ar-matrix isolation environment, providing reference data in the 4000–450 cm^–1^ (2.5–22 μm) range to support the analysis of laboratory and astronomical observations of formamide-containing interstellar ices. Spectral features arising from formamide complexation with water were analyzed with the aid of GVPT2 computations exploiting the reduced-dimensionality scheme. The new experiments, supported by calculations, enabled the identification of the elusive second-stable formamide–water complex, FAW-Ib, which is characterized by an O–H···O hydrogen bond. This complex was not identified in any previous MI-FTIR studies,^47^ although it was observed in the gas phase.^43^

For the complexes previously observed by MI-FTIR, namely FAW-Ia and FAW-Ic, anharmonic calculations allowed the identification of additional bands and the proposal of revised spectral assignments. For all complexes, computational support enabled linking characteristic band shifts to specific hydrogen bonds, N–H­(FA)···O­(W) and/or O–H­(W)···O­(FA). Notably, in all cases (also for the characteristic C=O stretching), the magnitude of the shifts due to the intermolecular hydrogen bonds agrees well between computations and experiment. This indicates that anharmonic calculations can also be exploited to analyze complex spectral patterns of multicomponent ices.

The experimental data were also used to assess the accuracy of different computational approaches and to propose computational strategies applicable to larger and more complex weakly bound aggregates. In this respect, comparison between counterpoise-corrected (CP) and uncorrected (NCP) calculations showed very good agreement with each other for both structures and spectra, with NCP performing slightly better for structural parameters. This outcome suggests that NCP computations can be confidently applied to larger adducts.

Overall, a very good agreement with experiment was obtained using reduced-dimensionality GVPT2 computations at the revDSD and hybrid revDSD/B3D3 levels. These results point out that, for the mid-IR region and the most intense transitions, RD-GVPT2 models, that exclude large-amplitude motions from the perturbative treatment, represent a feasible and reliable tool for anharmonic calculations of weakly bound systems.

## Supplementary Material





## Notes

Tables reporting the structural parameters of the FAW complexes computed at different levels of theory (Tables S1–S3); tables reporting the fundamental wavenumbers and IR intensities of the FAW complexes computed at different levels of theory (Tables S4–S6); tables reporting all experimental bands assigned to the FAW complexes along with their computed counterparts (Table S7) (PDF)
Files DAT of the experimental MI-FTIR spectra for FA/H_2_O/Ar mixture and for monomers (FA/Ar and H_2_O/Ar), deposited at 15 K, recorded at 10 K; the data set additionally contains spectra of the FA/H_2_O/Ar mixture recorded at 10 K after annealing the sample at 30 K for 5 min; files DAT of the RD-GVPT2 theoretical spectra of the FAW complexes and monomers (FA and W), computed at the NCP-revDSD level, with stick spectra convoluted with a HWHM of 1 cm^–1^ (ZIP)
