# Supplementary material for: Spectroscopic Features of Weak Intermolecular Interactions: IR Spectrum of the Formamide–Water Complex Revisited
Source: J Phys Chem A. 2026 Jul 31;130(33):6528–42. doi: 10.1021/acs.jpca.6c02029 (PMC13501956; doi:10.1021/acs.jpca.6c02029)
Supplement: Supplementary file 1 [file jp6c02029_si_001.pdf]

# Supporting Information for "Spectroscopic Features of Weak Intermolecular Interactions: IR Spectrum of Formamide-Water Complex Revisited"

Justyna Krupa,† Silvia Alessandrini,*,‡ Cristina Puzzarini,‡ and Malgorzata Biczysko*,†

†*Faculty of Chemistry, University of Wroclaw, F. Joliot-Curie 14, 50-383 Wroclaw, Poland*

‡*Dipartimento di Chimica "Giacomo Ciamician", Università di Bologna, Via P. Gobetti 85, 40129 Bologna, Italy*

E-mail: silvia.alessandrini7@unibo.it; malgorzata.biczysko@uwr.edu.pl

# Structural parameters for FWA complexes

**Table S1: CP and NCP structures of the formamide-water complex (FAW-Ia) at different levels of theory. For comparison, a partial semi-experimental equilibrium structure is also reported.[1]**

| Parameter | B3 | | revDSD | | $r_e^{SE}$ |
|---|---|---|---|---|---|
| | CP | NCP | CP | NCP | |
| C1O2 | 1.2225 | 1.2226 | 1.2247 | 1.2250 | 1.2225 |
| C1N3 | 1.3449 | 1.3448 | 1.3475 | 1.3471 | 1.3432 |
| H4C1 | 1.1006 | 1.1006 | 1.1003 | 1.1002 | 1.0943 |
| N3H5 | 1.0145 | 1.0146 | 1.0130 | 1.0133 | 1.0141 |
| N3H6 | 1.0036 | 1.0036 | 1.0035 | 1.0035 | 1.0168 |
| O2H7 | 1.8786 | 1.8756 | 1.9124 | 1.8917 | |
| O8H7 | 0.9776 | 0.9778 | 0.9744 | 0.9753 | 0.9728 |
| O8H9 | 0.9604 | 0.9604 | 0.9599 | 0.9600 | 0.9569 |
| O8H5 | 2.0616 | 2.0592 | 2.0773 | 2.0612 | 2.151(1) |
| O2C1N3 | 124.96 | 124.95 | 124.95 | 124.94 | 124.51 |
| H4C1O2 | 121.40 | 121.40 | 121.51 | 121.48 | 122.17 |
| H5N3C1 | 119.07 | 119.05 | 119.24 | 119.16 | 120.14 |
| H6N3C1 | 120.56 | 120.56 | 120.29 | 120.28 | 119.61 |
| C1O2H7 | 107.40 | 107.37 | 107.45 | 107.19 | |
| O2H7O8 | 151.47 | 151.53 | 150.67 | 151.22 | |
| H9O8H7 | 106.49 | 106.47 | 105.82 | 105.83 | 105.77 |
| O8H5N3 | 136.79 | 136.77 | 137.14 | 136.97 | 132.55(5) |
| H4C1O2N3 | -179.93 | -179.93 | -179.90 | -179.90 | |
| H5N3C1O2 | 0.15 | 0.13 | 0.39 | 0.37 | |
| H6N3C1O2 | 179.20 | 179.21 | 178.98 | 178.99 | |
| H7O2C1N3 | -2.09 | -2.06 | -2.39 | -2.35 | |
| O8H7O2C1 | -3.18 | -3.14 | -3.52 | -3.53 | |
| H9O8H7C1 | 142.22 | 141.84 | 144.85 | 144.04 | |
| $A_0$ | 11186.04 [-0.37][a] | 11185.93 [-0.37] | 11146.24 [-0.73] | 11188.46 [-0.35] | |
| $B_0$ | 4562.24[-0.54] | 4570.82 [-0.35] | 4489.69 [-2.12] | 4579.16 [-0.17] | |
| $C_0$ | 3244.53 [-0.44] | 3249.02 [-0.30] | 3203.49 [-1.70] | 3238.19 [-0.63] | |

[a] Relative deviation (%) with respect to experimental rotational constants of Ref. 2 are reported in square brackets.

**Table S2: CP and NCP structures of the formamide-water complex (FAW-Ib) at different levels of theory.**

| Parameter | B3 | | revDSD | |
|---|---|---|---|---|
| | CP | NCP | CP | NCP |
| C1O2 | 1.2185 | 1.2186 | 1.2215 | 1.2217 |
| C1N3 | 1.3487 | 1.3487 | 1.3508 | 1.3506 |
| H4C1 | 1.0999 | 1.0999 | 1.0998 | 1.0997 |
| N3H5 | 1.0069 | 1.0069 | 1.0063 | 1.0064 |
| N3H6 | 1.0040 | 1.0040 | 1.0037 | 1.0037 |
| O2H7 | 1.8811 | 1.8781 | 1.9179 | 1.8993 |
| O8H7 | 0.9737 | 0.9739 | 0.9713 | 0.9719 |
| O8H9 | 0.9602 | 0.9602 | 0.9598 | 0.9597 |
| O2C1N3 | 124.30 | 124.31 | 124.20 | 124.20 |
| H4C1O2 | 122.07 | 122.06 | 122.12 | 122.09 |
| H5N3C1 | 119.65 | 119.66 | 119.52 | 119.53 |
| H6N3C1 | 121.10 | 121.10 | 120.94 | 120.93 |
| C1O2H7 | 103.62 | 103.55 | 101.03 | 100.74 |
| O2H7O8 | 158.44 | 158.41 | 155.17 | 155.40 |
| H9O8H7 | 105.64 | 105.62 | 105.09 | 105.10 |
| H4C1O2N3 | 179.95 | -179.94 | 179.92 | 179.92 |
| H5N3C1O2 | -0.40 | 0.42 | -0.49 | -0.51 |
| H6N3C1O2 | -179.60 | 179.58 | -179.47 | -179.46 |
| H7O2C1N3 | 177.70 | -177.63 | 178.16 | 178.07 |
| O8H7O2C1 | -2.42 | 2.46 | -2.32 | -2.39 |
| H9O8H7O2 | 156.46 | -156.06 | 162.07 | 161.87 |
| $A_0$ | 28659.46 [9.51][a] | 28616.32 [9.34] | 26421.26 [ 0.96] | 26346.56 [0.67] |
| $B_0$ | 2602.37 [-3.00] | 2607.89 [-2.80] | 2660.60 [-0.83] | 2688.02 [0.19] |
| $C_0$ | 2374.85 [-2.42] | 2379.26 [-2.84] | 2403.07 [-1.26] | 2424.90 [-0.37] |

[a] Relative deviation (%) with respect to experimental rotational constants of Ref. 2 are reported in square brackets.

**Table S3: CP and NCP structures of the formamide-water complex (FAW-Ic) at different levels of theory.**

| Parameter | B3 | | revDSD | |
|---|---|---|---|---|
| | CP | NCP | CP | NCP |
| C1O2 | 1.2145 | 1.2145 | 1.2178 | 1.2178 |
| C1N3 | 1.3512 | 1.3512 | 1.3533 | 1.3531 |
| H4C1 | 1.1043 | 1.1043 | 1.1032 | 1.1033 |
| N3H5 | 1.0065 | 1.0065 | 1.0061 | 1.0061 |
| N3H6 | 1.0101 | 1.0101 | 1.0089 | 1.0091 |
| O8H6 | 2.0065 | 2.0030 | 2.0326 | 2.0140 |
| O8H7 | 0.9618 | 0.9619 | 0.9611 | 0.9612 |
| O2C1N3 | 125.51 | 125.51 | 125.31 | 125.34 |
| H4C1O2 | 122.23 | 122.23 | 122.33 | 122.31 |
| H5N3C1 | 118.84 | 118.84 | 118.73 | 118.71 |
| H6N3C1 | 121.32 | 121.31 | 121.13 | 121.12 |
| O8H6N3 | 176.62 | 176.61 | 175.50 | 175.79 |
| H6O8H7 | 124.76 | 125.14 | 126.35 | 125.90 |
| H9O8H7 | 105.84 | 105.84 | 105.16 | 125.90 |
| H7O8H6N3 | -76.18 | -77.31 | -80.41 | -78.63 |
| H9O8H6N3 | 76.18 | 77.31 | 80.41 | 78.63 |
| $B_0$ | 2072.65 [-0.58][a] | 2074.71 [-0.48] | 2057.79 [-1.29] | 2071.78 [-0.62] |
| $C_0$ | 1968.92 [0.98 ] | 1971.01 [-1.09] | 1953.24 [0.18] | 1966.42 [0.85] |

[a] Relative deviation (%) with respect to experimental rotational constants of Ref. 2 are reported in square brackets.

# Vibrational features of FWA complexes

**Table S4: Fundamental wavenumbers[a] and IR intensities of the formamide-water complex FAW-Ia at different levels of theory. Wavenumbers in cm$^{-1}$, IR intensities in km/mol.**

| Mode[b] | B3D3 | | | | | | | | revDSD | | | | | | | | | | | | revDSD/B3D3 | | Assignment[c] |
|---|---|---|---|---|---|---|---|---|---|---|---|---|---|---|---|---|---|---|---|---|---|---|
| | CP | | | | NCP | | | | CP | | | | NCP | | | | | | NCP | | |
| | $\omega$ | $IR_{harm}$ | $\nu$ | $IR_{anh}$ | $\omega$ | $IR_{harm}$ | $\nu$ | $IR_{anh}$ | $\omega$ | $IR_{harm}$ | $\nu$ | $IR_{anh}$ | $\omega$ | $IR_{harm}$ | $\nu^{NRD}$ | $IR^{NRD}_{anh}$ | $\nu$ | $IR_{anh}$ | $\nu$ | $IR_{anh}$ | |
| $\nu_1$ | 3877 | 70 | 3692 | 49 | 3876 | 69 | 3692 | 48 | 3905 | 81 | 3717 | 59 | 3903 | 79 | 3734 | 55 | 3717 | 56 | 3727 | 47 | $\nu_3$(W) |
| $\nu_2$ | 3691 | 93 | 3521 | 72 | 3691 | 93 | 3521 | 72 | 3727 | 107 | 3556 | 88 | 3726 | 106 | 3553 | 66 | 3556 | 86 | 3563 | 71 | $\nu_1$(FA) |
| $\nu_3$ | 3541 | 537 | 3356 | 561 | 3538 | 541 | 3353 | 555 | 3615 | 395 | 3414 | 458 | 3599 | 436 | 3441 | 356 | 3396 | 511 | 3419 | 579 | $\nu_1$(W) |
| $\nu_4$ | 3472 | 44 | 3276 | 37 | 3471 | 41 | 3273 | 43 | 3523 | 79 | 3356 | 50 | 3517 | 67 | 3366 | 55 | 3347 | 32 | 3330 | 15 | $\nu_2$(FA) |
| $\nu_5$ | 2979 | 93 | 2845 | 69 | 2978 | 93 | 2846 | 68 | 3021 | 84 | 2881 | 70 | 3022 | 84 | 2881 | 70 | 2882 | 71 | 2884 | 80 | $\nu_3$(FA) |
| $\nu_6$ | 1756 | 393 | 1718 | 393 | 1756 | 394 | 1717 | 391 | 1768 | 387 | 1728 | 387 | 1767 | 387 | 1734 | 346 | 1727 | 388 | 1729 | 396 | $\nu_4$(FA) |
| $\nu_7$ | 1646 | 132 | 1585 | 28 | 1646 | 130 | 1585 | 28 | 1661 | 138 | 1595 | 53 | 1662 | 139 | 1614 | 135 | 1594 | 55 | 1596 | 47 | $\nu_2$(W) |
| $\nu_8$ | 1620 | 38 | 1563 | 124 | 1620 | 37 | 1563 | 121 | 1630 | 29 | 1577 | 99 | 1628 | 27 | 1592 | 22 | 1576 | 92 | 1575 | 100 | $\nu_5$(FA) |
| $\nu_9$ | 1418 | 16 | 1388 | 12 | 1418 | 16 | 1388 | 13 | 1424 | 15 | 1395 | 13 | 1424 | 16 | 1395 | 9 | 1395 | 14 | 1395 | 15 | $\nu_6$(FA) |
| $\nu_{10}$ | 1318 | 103 | 1279 | 91 | 1318 | 103 | 1280 | 93 | 1325 | 105 | 1291 | 109 | 1326 | 104 | 1286 | 93 | 1292 | 108 | 1293 | 107 | $\nu_7$(FA) |
| $\nu_{11}$ | 1089 | 2 | 1068 | 2 | 1090 | 2 | 1068 | 2 | 1089 | 2 | 1068 | 2 | 1090 | 2 | 1060 | 3 | 1068 | 2 | 1069 | 2 | $\nu_8$(FA) |
| $\nu_{12}$ | 1051 | 4 | 1026 | 3 | 1052 | 4 | 1027 | 3 | 1060 | 5 | 1034 | 4 | 1060 | 5 | 1037 | 3 | 1035 | 4 | 1036 | 3 | $\nu_{10}$(FA) |
| $\nu_{13}$ | 762 | 71 | 737 | 39 | 764 | 71 | 739 | 38 | 748 | 55 | 717 | 32 | 754 | 58 | 700 | 30 | 723 | 32 | 729 | 41 | $\nu_{11}$(FA) |
| $\nu_{14}$ | 687 | 127 | 628 | 150 | 690 | 124 | 628 | 149 | 652 | 126 | 579 | 97 | 667 | 122 | 527 | 78 | 617 | 79 | 611 | 104 | |
| $\nu_{15}$ | 612 | 10 | 605 | 21 | 612 | 10 | 605 | 21 | 607 | 10 | 619 | 64 | 608 | 10 | 604 | 9 | 589 | 82 | 598 | 64 | $\nu_9$(FA) |
| $\nu_{16}$ | 436 | 160 | | | 436 | 157 | | | 416 | 146 | | | 422 | 139 | 552 | 79 | | | | | |
| $\nu_{17}$ | 404 | 90 | | | 405 | 89 | | | 367 | 112 | | | 372 | 116 | 315 | 90 | | | | | |
| $\nu_{18}$ | 209 | 57 | | | 210 | 56 | | | 198 | 53 | | | 203 | 51 | 323 | 680 | | | | | |
| $\nu_{19}$ | 176 | 26 | | | 177 | 25 | | | 169 | 26 | | | 173 | 24 | 172 | 29 | | | | | |
| $\nu_{20}$ | 170 | 34 | | | 170 | 34 | | | 160 | 40 | | | 161 | 38 | 8 | 0 | | | | | |
| $\nu_{21}$ | 144 | 77 | | | 144 | 77 | | | 134 | 77 | | | 135 | 81 | 178 | 78 | | | | | |

[a] Harmonic and anharmonic wavenumbers are denoted as $\omega$ and $\nu$, respectively. Anharmonic computations are at the RD-GVPT2 level, except the $\nu$NRD ones which do not exploit the reduced dimensionality model.[b] Modes listed in the spectroscopic order according to the $C_1$ symmetry. [c] Intramolecular vibrations of formamide and water monomers within the FAW complex are labelled using (FA) and (W), respectively.

**Table S5: Fundamental wavenumbers[a] and IR intensities of the formamide-water complex FAW-Ib at different levels of theory. Wavenumbers in cm$^{-1}$, IR intensities in km/mol.**

| Mode[b] | B3D3 | | | | | | | | revDSD | | | | | | | | | | revDSD/B3D3 | | Assignment[c] |
|---|---|---|---|---|---|---|---|---|---|---|---|---|---|---|---|---|---|---|---|---|---|
| | CP | | | | NCP | | | | CP | | | | NCP | | | | | | NCP | | |
| | $\omega$ | IR$_{harm}$ | $\nu$ | IR$_{anh}$ | $\omega$ | IR$_{harm}$ | $\nu$ | IR$_{anh}$ | $\omega$ | IR$_{harm}$ | $\nu$ | IR$_{anh}$ | $\omega$ | IR$_{harm}$ | $\nu^{NRD}$ | IR$_{anh}^{NRD}$ | $\nu$ | IR$_{anh}$ | $\nu$ | IR$_{anh}$ | |
| $\nu_1$ | 3880 | 80 | 3704 | 53 | 3879 | 79 | 3704 | 52 | 3909 | 92 | 3729 | 63 | 3908 | 89 | 3734 | 106 | 3729 | 61 | 3741 | 51 | $\nu_3$(W) |
| $\nu_2$ | 3715 | 52 | 3536 | 46 | 3715 | 52 | 3536 | 46 | 3752 | 58 | 3576 | 53 | 3752 | 58 | 3567 | 45 | 3576 | 53 | 3580 | 46 | $\nu_1$(FA) |
| $\nu_3$ | 3603 | 485 | 3402 | 513 | 3600 | 479 | 3398 | 518 | 3666 | 377 | 3481 | 268 | 3654 | 394 | 3512 | 289 | 3474 | 179 | 3485 | 202 | $\nu_1$(W) |
| $\nu_4$ | 3578 | 76 | 3436 | 41 | 3578 | 81 | 3437 | 35 | 3609 | 69 | 3456 | 183 | 3608 | 74 | 3444 | 72 | 3452 | 288 | 3447 | 350 | $\nu_2$(FA) |
| $\nu_5$ | 2995 | 37 | 2867 | 30 | 2995 | 37 | 2867 | 30 | 3036 | 33 | 2902 | 29 | 3037 | 32 | 2901 | 31 | 2903 | 28 | 2904 | 33 | $\nu_3$(FA) |
| $\nu_6$ | 1763 | 467 | 1726 | 452 | 1762 | 467 | 1726 | 452 | 1770 | 438 | 1734 | 439 | 1770 | 436 | 1743 | 253 | 1733 | 439 | 1735 | 460 | $\nu_4$(FA) |
| $\nu_7$ | 1658 | 96 | 1587 | 84 | 1658 | 95 | 1587 | 83 | 1671 | 113 | 1599 | 86 | 1673 | 114 | 1615 | 251 | 1602 | 84 | 1603 | 71 | $\nu_2$(W) |
| $\nu_8$ | 1622 | 48 | 1574 | 49 | 1622 | 48 | 1574 | 49 | 1629 | 40 | 1573 | 22 | 1629 | 40 | 1589 | 33 | 1580 | 37 | 1580 | 45 | $\nu_5$(FA) |
| $\nu_9$ | 1425 | 14 | 1396 | 13 | 1425 | 14 | 1396 | 13 | 1432 | 14 | 1404 | 13 | 1432 | 14 | 1409 | 11 | 1404 | 13 | 1403 | 13 | $\nu_6$(FA) |
| $\nu_{10}$ | 1287 | 118 | 1273 | 86 | 1287 | 118 | 1273 | 84 | 1297 | 124 | 1279 | 100 | 1297 | 124 | 1278 | 70 | 1280 | 99 | 1281 | 94 | $\nu_7$(FA) |
| $\nu_{11}$ | 1069 | 2 | 1047 | 1 | 1069 | 2 | 1047 | 1 | 1070 | 2 | 1052 | 2 | 1071 | 2 | 1072 | 6 | 1053 | 2 | 1049 | 1 | $\nu_8$(FA) |
| $\nu_{12}$ | 1058 | 3 | 1035 | 3 | 1058 | 3 | 1035 | 3 | 1066 | 4 | 1038 | 3 | 1066 | 4 | 1031 | 3 | 1039 | 3 | 1043 | 3 | $\nu_{10}$(FA) |
| $\nu_{13}$ | 653 | 22 | 614 | 22 | 653 | 22 | 614 | 23 | 652 | 17 | 614 | 16 | 652 | 17 | 624 | 3 | 615 | 18 | 614 | 24 | $\nu_{11}$(FA) |
| $\nu_{14}$ | 617 | 80 | 654 | 11 | 620 | 79 | 652 | 11 | 590 | 40 | 689 | 15 | 599 | 61 | 252 | 140 | 674 | 87 | 640 | 101 | |
| $\nu_{15}$ | 577 | 42 | 485 | 100 | 577 | 41 | 486 | 99 | 569 | 76 | 407 | 80 | 573 | 52 | 599 | 36 | 446 | 11 | 479 | 8 | $\nu_9$(FA) |
| $\nu_{16}$ | 404 | 139 | | | 404 | 136 | | | 394 | 147 | | | 402 | 146 | 397 | 205 | | | | | |
| $\nu_{17}$ | 313 | 200 | | | 313 | 199 | | | 251 | 209 | | | 252 | 209 | 441 | 123 | | | | | |
| $\nu_{18}$ | 190 | 16 | | | 192 | 16 | | | 184 | 15 | | | 189 | 15 | 166 | 16 | | | | | |
| $\nu_{19}$ | 71 | 12 | | | 71 | 12 | | | 75 | 17 | | | 76 | 21 | 100 | 5814 | | | | | |
| $\nu_{20}$ | 62 | 70 | | | 63 | 64 | | | 68 | 22 | | | 70 | 18 | 40 | 346 | | | | | |
| $\nu_{21}$ | 59 | 65 | | | 59 | 70 | | | 47 | 108 | | | 46 | 107 | -203 | 12420 | | | | | |

[a] Harmonic and anharmonic wavenumbers are denoted as $\omega$ and $\nu$, respectively. Anharmonic computations are at the RD-GVPT2 level, except the $\nu$NRD ones which do not exploit the reduced dimensionality model.[b] Modes listed in the spectroscopic order according to the $C_1$ symmetry. [c] Intramolecular vibrations of formamide and water monomers within the FAW complex are labelled using (FA) and (W), respectively.

Table S6: **Fundamental wavenumbers[a] and IR intensities of the formamide-water complex FAW-Ic at different levels of theory. Wavenumbers in $cm^{-1}$, IR intensities in km/mol.**

| Mode[b] | B3D3 | | | | | | | | revDSD | | | | | | | | | | revDSD/B3D3 | | Assignment[c] |
|---|---|---|---|---|---|---|---|---|---|---|---|---|---|---|---|---|---|---|---|---|---|
| | CP | | | | NCP | | | | CP | | | | NCP | | | | | | NCP | | |
| | $\omega$ | $IR_{harm}$ | $\nu$ | $IR_{anh}$ | $\omega$ | $IR_{harm}$ | $\nu$ | $IR_{anh}$ | $\omega$ | $IR_{harm}$ | $\nu$ | $IR_{anh}$ | $\omega$ | $IR_{harm}$ | $\nu^{NRD}$ | $IR^{NRD}_{anh}$ | $\nu$ | $IR_{anh}$ | $\nu$ | $IR_{anh}$ | |
| $\nu_1$ | 3804 | 15 | 3651 | 13 | 3804 | 13 | 3650 | 11 | 3826 | 14 | 3670 | 11 | 3824 | 13 | 3651 | 12 | 3669 | 10 | 3676 | 11 | $\nu_1$(W) |
| $\nu_2$ | 3671 | 135 | 3488 | 115 | 3671 | 134 | 3488 | 115 | 3714 | 148 | 3534 | 127 | 3712 | 149 | 3543 | 60 | 3533 | 129 | 3532 | 116 | $\nu_1$(FA) |
| $\nu_3$ | 3518 | 268 | 3357 | 238 | 3518 | 267 | 3357 | 238 | 3561 | 234 | 3408 | 243 | 3557 | 247 | 3417 | 155 | 3404 | 260 | 3397 | 192 | $\nu_2$(FA) |
| $\nu_4$ | 2934 | 98 | 2711 | 43 | 2935 | 98 | 2711 | 42 | 2986 | 87 | 2855 | 70 | 2985 | 88 | 2860 | 64 | 2854 | 70 | 2857 | 78 | $\nu_3$(FA) |
| $\nu_5$ | 1776 | 475 | 1740 | 329 | 1776 | 475 | 1740 | 338 | 1780 | 462 | 1744 | 424 | 1780 | 462 | 1742 | 315 | 1741 | 346 | 1744 | 334 | $\nu_4$(FA) |
| $\nu_6$ | 1636 | 38 | 1597 | 36 | 1636 | 39 | 1596 | 35 | 1647 | 75 | 1581 | 67 | 1646 | 74 | 1590 | 51 | 1583 | 80 | 1604 | 35 | $\nu_2$(W) |
| $\nu_7$ | 1628 | 79 | 1566 | 77 | 1628 | 78 | 1566 | 79 | 1641 | 33 | 1594 | 41 | 1643 | 34 | 1601 | 81 | 1595 | 34 | 1581 | 77 | $\nu_5$(FA) |
| $\nu_8$ | 1416 | 10 | 1386 | 9 | 1416 | 10 | 1386 | 9 | 1420 | 10 | 1389 | 9 | 1420 | 9 | 1389 | 10 | 1389 | 8 | 1390 | 9 | $\nu_6$(FA) |
| $\nu_9$ | 1281 | 104 | 1258 | 104 | 1281 | 104 | 1258 | 104 | 1291 | 107 | 1261 | 112 | 1292 | 107 | 1266 | 107 | 1262 | 111 | 1270 | 107 | $\nu_7$(FA) |
| $\nu_{10}$ | 1083 | 5 | 1057 | 7 | 1083 | 5 | 1057 | 7 | 1082 | 5 | 1059 | 5 | 1084 | 5 | 1056 | 8 | 1062 | 5 | 1056 | 7 | $\nu_8$(FA) |
| $\nu_{11}$ | 584 | 16 | 582 | 17 | 584 | 16 | 582 | 17 | 580 | 16 | 575 | 16 | 581 | 16 | 579 | 15 | 577 | 16 | 580 | 17 | $\nu_9$(FA) |
| $\nu_{12}$ | 152 | 7 | | | 153 | 7 | | | 147 | 6 | | | 150 | 7 | 139 | 26 | | | | | |
| $\nu_{13}$ | 106 | 238 | | | 100 | 234 | | | 88 | 236 | | | 103 | 233 | -492 | 12925 | | | | | |
| $\nu_{14}$ | 50 | 5 | | | 50 | 4 | | | 46 | 4 | | | 47 | 4 | 23 | 19 | | | | | |
| $\nu_{15}$ | 3905 | 87 | 3724 | 84 | 3905 | 88 | 3724 | 83 | 3938 | 85 | 3755 | 81 | 3937 | 86 | 3751 | 63 | 3752 | 82 | 3762 | 83 | $\nu_3$(W) |
| $\nu_{16}$ | 1045 | 2 | 1021 | 2 | 1045 | 2 | 1022 | 2 | 1053 | 4 | 1030 | 4 | 1053 | 4 | 1031 | 3 | 1029 | 4 | 1030 | 2 | $\nu_{10}$(FA) |
| $\nu_{17}$ | 740 | 3 | 697 | 2 | 740 | 3 | 697 | 2 | 730 | 1 | 689 | 1 | 736 | 2 | 665 | 0 | 695 | 2 | 696 | 2 | $\nu_{11}$(FA) |
| $\nu_{18}$ | 488 | 244 | 481 | 230 | 489 | 244 | 481 | 229 | 446 | 260 | 446 | 260 | 453 | 256 | 470 | 211 | 453 | 256 | 455 | 228 | |
| $\nu_{19}$ | 178 | 8 | | | 179 | 9 | | | 159 | 5 | | | 165 | 4 | 139 | 33 | | | | | |
| $\nu_{20}$ | 97 | 12 | | | 99 | 11 | | | 101 | 7 | | | 102 | 9 | -166 | 1 | | | | | |
| $\nu_{21}$ | 63 | 0.2 | | | 64 | 0.1 | | | 58 | 0.1 | | | 60 | 0.01 | -76 | 7 | | | | | |

[a] Harmonic and anharmonic wavenumbers are denoted as $\omega$ and $\nu$, respectively. Anharmonic computations are at the RD-GVPT2 level, except the $\nu$NRD ones which do not exploit the reduced dimensionality model.[b] Modes listed in the spectroscopic order according to the $C_s$ symmetry. [c] Intramolecular vibrations of formamide and water monomers within the FAW complex are labelled using (FA) and (W), respectively.

**Table S7: Experimental MI-FTIR data (this work) for the formamide and water monomers[a] vibrations within the formamide-water FAW-Ia, FAW-Ib, and FAW-Ic complexes compared with the RD-GVPT2 NCP-revDSD computations, wavenumbers in $cm^{-1}$.**

| Assignment[b] | FAW-Ia | | | | FAW-Ib | | | | FAW-Ic | | | |
|---|---|---|---|---|---|---|---|---|---|---|---|---|
| | $\nu$ | $\Delta$ | Exp. | $\Delta$ | $\nu$ | $\Delta$ | Exp. | $\Delta$ | $\nu$ | $\Delta$ | Exp. | $\Delta$ |
| $\nu_3$(W) | 3717 | -36 | 3706 | -30 | 3729 | -24 | 3715 | -21 | 3752 | -1 | 3726 | -10 |
| $\nu_1$(W) | 3396 | -255 | 3430 | -208 | 3474 | -177 | 3514 | -124 | 3669 | 18 | 3640 | 2 |
| $\nu_1$(FA) | 3556 | -31 | 3532 | -15 | 3576 | -11 | 3555 | 8 | 3533 | -54 | 3524 | -23 |
| $\nu_2$(FA) | 3347 | -113 | 3355 | -73 | 3452 | -8 | 3465 | 38 | 3404 | -56 | 3388.6 | -38 |
| $\nu_4$(FA) | 1727 | -25 | 1717 | -21 | 1733 | -19 | 1725 | -13 | 1741 | -11 | 1731.3 | -7 |
| $\nu_2$(W) | 1594 | 9 | 1598 | 4 | 1602 | 12 | 1601 | 12 | 1583 | -7 | 1590 | 1 |
| $\nu_5$(FA) | 1576 | -1 | 1586 | 10 | 1581 | 4 | 1580 | 4 | 1595 | 18 | 1594 | 18 |
| $\nu_7$(FA) | 1292 | 33 | 1281 | 19 | 1280 | 21 | 1287 | 25 | 1262 | 3 | 1273 | 11 |

[a] Anharmonic wavenumbers are denoted as $\nu$, while $\Delta$ refers to the shift with respect to monomer. [b] Intramolecular vibrations of the formamide and water monomers within the FAW complex are labelled as (FA) and (W), respectively.

# References

(1) Puzzarini, C.; Spada, L.; Alessandrini, S.; Barone, V. The challenge of non-covalent interactions: theory meets experiment for reconciling accuracy and interpretation. *J. Phys. Condens. Matter* **2020**, *32*, 343002.

(2) Blanco, S.; López, J. C.; Lesarri, A.; Alonso, J. L. Microsolvation of Formamide: A Rotational Study. *J. Am. Chem. Soc.* **2006**, *128*, 12111–12121.
